# Genomic complexity in advanced gastric and esophageal adenocarcinomas: a case report of rare *WDR11*-*AS1*-*FGFR2* fusions

**DOI:** 10.3389/fonc.2025.1686070

**Published:** 2025-11-20

**Authors:** Eric Mehlhaff, Dustin A. Deming, Jeremy D. Kratz, Khaldun Obeidat, Lauren Welch, Nataliya Uboha

**Affiliations:** 1 Department of Medicine, Division of Hematology, Medical Oncology and Palliative Care, School of Medicine and Public Health, University of Wisconsin, Madison WI, United States; 2 Carbone Cancer Center, University of Wisconsin, Madison, WI, United States; 3 William S. Middleton Memorial Veterans Hospital, Madison, WI, United States; 4 Department of Hospital Medicine, School of Medicine and Public Health, University of Wisconsin, Madison, WI, United States; 5 Guardant Health, Palo Alto, CA, United States

**Keywords:** gastric cancer, biomarker, WDR11-AS1, liquid biopsy, FGFR2 fusion

## Abstract

Fibroblast growth factor receptor 2 (FGFR2) alterations represent an emerging therapeutic target in gastroesophageal adenocarcinoma (GEA). Although FGFR2 amplifications and overexpression have been associated with poor prognosis and therapeutic resistance, the clinical significant FGFR2 fusions, which are exceedingly rare, is unknown. Herein we describe two cases of advanced gastroesophageal and gastric adenocarcinomas characterized by aggressive disease course, rapid progression despite standard first-line chemoimmunotherapy, and the presence of high-level FGFR2 amplification with concurrent WDR11-AS1–FGFR2 fusion detected by circulating tumor DNA (ctDNA) analysis. These cases highlight the genomic complexity and aggressive behavior of FGFR2-driven GEA, underscored by coexisting genetic alterations. The findings emphasize the importance of comprehensive genomic profiling, including both tissue and liquid. in order to capture intratumoral heterogeneity and evolving molecular events. Further investigation of FGFR2 fusion biology and combinatorial therapeutic strategies is warranted to address the clinical challenge of biomarker overlap and treatment resistance in GEA.

## Introduction

Fibroblast growth factor receptors (FGFRs) are a family of proteins involved in key cellular processes such as proliferation, differentiation, and survival (1). *FGFR* aberrations are observed across a wide range of cancers, with up to 7.1% of all tumors harboring mutations, fusions, or gene amplifications that result in abnormal *FGFR* signaling. The prevalence of *FGFR* alterations is reported in 17-26% of gastric carcinomas and approximately 13% of esophageal carcinomas (1–4). Among gastric tumors, *FGFR2* is the most frequently affected subtype, with mutations reported in 3-11% of cases, rearrangements in about 3%, and amplifications in roughly 9% of tumors (5, 6). Alterations were less commonly involving *FGFR1* (3.5%), *FGFR3* (<1%), and *FGFR4* (<1%) (6).

*FGFR2* gene amplification, the most common alteration in gastrointestinal cancers, is associated with poor clinical outcomes, including lymph node metastasis, poorly differentiated histology, and reduced overall survival compared to non-amplified tumors (5, 7). *FGFR2* amplification has also been linked to resistance against standard chemotherapy, particularly platinum-based and fluoropyrimidine regimens (8, 9). As a result, there is a growing interest in *FGFR2* as a potential therapeutic target to improve patient outcomes.

Emerging biomarkers in gastric and gastroesophageal junction (GEJ) adenocarcinomas include FGFR2b overexpression, identified via immunohistochemistry (IHC), and *FGFR2* gene amplification. Results from the phase II FIGHT trial demonstrated promising activity of bemarituzumab, an FGFR2b-targeting monoclonal antibody, when combined with FOLFOX6 as a first-line treatment for HER2-negative advanced gastric/GEA. Clinical benefits were more evident in patients with FGFR2b overexpression ≥10%, suggesting a threshold effect for therapeutic efficacy (PFS: HR 0.43; 95% CI 0.26–0.73; OS: HR 0.52; 95% CI 0.31–0.85) (10). These results await confirmation in the phase III FORTITUDE-101 trial.

Targeting *FGFR* alterations with tyrosine kinase inhibitors (TKI) in upper gastrointestinal cancers has also been explored. Erdafitinib, a selective pan-FGFR TKI, was evaluated in a phase II tumor-agnostic trial involving patients with *FGFR*1–4 activating mutations or fusions. Among enrolled patients, 4% had esophageal cancer (*n* = 8) and 4% had gastric cancer (*n* = 8). In the subgroup analysis, the objective response rate (ORR) was 13%, while disease control was achieved in 38% of esophageal and 63% of gastric cancer patients. Notably, most responses were observed in individuals harboring *FGFR* fusions (11). Futibatinib, an irreversible inhibitor of FGFR1-4, also demonstrated modest antitumor effect (ORR 17.9%) among patients with *FGFR2*-amplified gastric and GEJ cancers (*n* = 28) in a recent phase II trial (12). These findings highlight the therapeutic potential of *FGFR2*-targeted strategies in gastroesophageal adenocarcinoma (GEA) malignancies. However, treatment options remain limited. Three *FGFR* inhibitors (erdafitinib, pemigatinib, futibatinib) are currently FDA-approved for *FGFR*2/3-alterations in cholangiocarcinoma and bladder cancer; however, none have been approved for gastric or gastroesophageal cancers, highlighting a critical need for drug development (1, 5).

**Figure 1:**
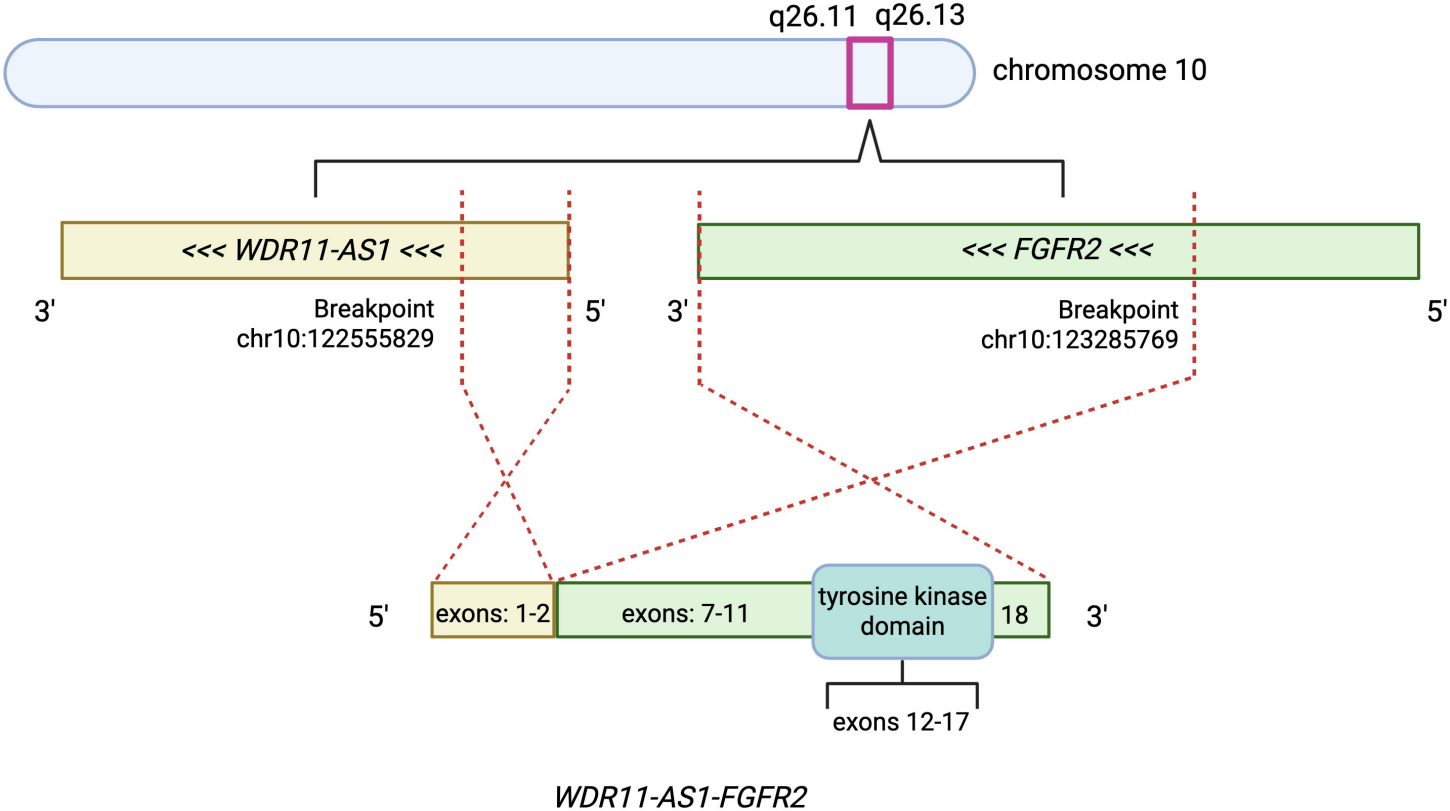
Schematic of the *WDR11-AS1–FGFR2* fusion on chromosome 10. An intrachromosomal rearrangement juxtaposes the *WDR11-AS1* promoter (5′ partner) to *FGFR2*, generating a chimeric transcript that joins *WDR11-AS1* exons 1–2 to *FGFR2* exons 7–18. Breakpoints (hg19/GRCh37; per Methods) are indicated at chr10:122,555,829 (*WDR11-AS1*) and chr10:123,285,769 (*FGFR2*). The fusion retains the entire *FGFR2* tyrosine-kinase domain (exons 12–17), highlighted on the schematic. Orientation (5′→3′) is shown; diagram not to scale. Created in BioRender. Welch, L. (2025) https://BioRender.com/tzlybag.

In this report, we present two cases of advanced gastric and esophageal/GEJ adenocarcinoma, respectively, in which both patients underwent circulating tumor DNA sequencing (ctDNA). The sequencing revealed the presence of *WDR11-AS1-FGFR2* fusions alongside significant gene amplifications, including high plasma copy numbers of *FGFR2*. A schematic diagram of this unique fusion is included (**Figure 1**), and genomic coordinates are based on the human reference genome assembly GRCh37 (hg19, Genome Reference Consortium Human Build 37, February 2009). The molecular genomics, clinical characteristics, treatment histories, and outcomes for these patients are presented. Additionally, the potential implications of these rare alterations in therapeutic decision-making and the utility of molecular profiling to detect informative biomarkers that may be challenging to detect via tissue alone given tumor evolution and heterogeneity is discussed.

## Case 1

A 46-year-old male presented with abdominal pain, early satiety, and a 25-pound weight loss over the course of three weeks. CT abdomen/pelvis revealed thickening of the distal esophagus with mass-like extension into the gastric fundus (measuring up to 6.6 x 3.0 cm), numerous lymph nodes, and several low-attenuating liver lesions. An endoscopic biopsy of the gastroesophageal mass demonstrated poorly differentiated adenocarcinoma with signet ring cell features. Biomarker testing showed HER2 3+ expression by IHC, PD-L1 combined positive score (CPS) of 1%, and intact nuclear expression of mismatch repair (MMR) proteins. A biopsy of the liver lesion confirmed poorly differentiated carcinoma, consistent with metastatic GEA.

The patient enrolled in a clinical trial evaluating a new chemotherapy backbone for advanced GEA and received NALIRIFOX (liposomal irinotecan, oxaliplatin, 5-fluorouracil), trastuzumab, and pembrolizumab. Despite initial disease response after three treatment cycles, his disease quickly progressed by the sixth month. Molecular testing of the tumor tissue at diagnosis revealed *ERBB2* gene amplification (estimated copy number of 6) and concurrent *FGFR2* amplification (estimated copy number of 128).

The patient underwent ctDNA profiling with Guardant360^®^ following progression on first-line therapy. The sequencing results included analysis of alterations in up to 83 genes, including amplifications and fusions, with notable findings of *FGFR2* amplification (plasma copy number of 83.1) and a *WDR11-AS1-FGFR2* fusion (2.4% variant allele frequency; **Table 1**). A schematic diagram of this specific fusion is depicted in **Figure 1**. Despite being enrolled in a clinical trial with a novel FGFR inhibitor as the next line of therapy, his cancer continued to progress rapidly. The patient ultimately succumbed to his disease nine months after the initial diagnosis.

**Table 1:** Summary of genomic findings detected by Guardant360^®^ at clinical progression.

Alteration	Mutation class	Variant allele frequency (VAF)	Plasma copy number
WDR11-AS1-FGFR2	Fusion	2.4%	
ERBB2	Amplification		4.8
PALB2	Single copy deletion		1.4
KRAS G12S	Missense	0.08%	
FGFR2	Amplification		83.1
EGFR	Amplification		2.3
AR	Amplification		1.3
TP53 R248W	Missense	55.3%	

## Case 2

A 19-year-old female presented with a 10-month history of progressive abdominal pain, nausea, and unintentional weight loss. Upon evaluation in the emergency department, cross-sectional imaging revealed extensive retroperitoneal and gastrohepatic ligament lymphadenopathy, diffuse gastric wall thickening, and nonspecific sclerotic foci throughout her skeleton. Esophagogastroduodenoscopy showed normal-appearing gastric mucosa; however, endoscopic ultrasound identified two large perigastric and gastrohepatic masses. Biopsy of a gastric lymph node confirmed poorly differentiated adenocarcinoma with signet ring cell features. Immunohistochemistry revealed intact MMR protein expression, PD-L1 CPS of 3%, and HER2 overexpression (IHC 3+). The patient initiated first-line palliative treatment with FOLFOX, trastuzumab, and pembrolizumab. Her early treatment course was complicated by severe cytopenia, likely related to extensive skeletal metastasis and suspected bone marrow involvement. Despite initial improvements in blood counts and cancer associated bone pain, restaging scans after 8 weeks of treatment showed disease progression, including further osteoblastic metastases and worsening lymphangitic carcinomatosis. Concurrently, the patient developed worsening respiratory symptoms and progressive bone pain.

At the initial oncology consultation, tissue-based molecular profiling using StrataNGS (13) identified *FGFR2*, *MYC*, and *CCNE1* gene amplifications, with the *FGFR2* amplification reaching a copy number of 241. Interestingly, no *ERBB2* gene alterations were detected in tumor tissue, despite positive IHC evaluation. Following progression on first-line therapy, ctDNA profiling with Guardant360^®^ CDx, which evaluates for mutations in 74 genes, identified *FGFR2* amplification at 82.6 copies and a *WDR11*-*AS1*–*FGFR2* fusion at 0.1% VAF (**Table 2**). In contrast to case 1, this fusion did not contain an active kinase domain; however, a reciprocal activating fusion (*FGFR2*–*WDR11*-*AS1*) could have been present but undetected. Further treatments, including clinical trial enrollment, were not possible due to significant disease progression, and she passed away 4 months after diagnosis.

**Table 2:** Summary of genomic findings detected by Guardant360^®^ CDx at clinical progression.

Alteration	Mutation class	Variant allele frequency (VAF)	Plasma copy number
WDR11-AS1-FGFR2	Fusion	0.1%	
TP53 R342*	Nonsense	37.5%	
FGFR2	Amplification		82.6
MYC	Amplification		8.0
CCNE1	Amplification		5.6
BRAF	Amplification		2.8

## Discussion

We report two rare, aggressive cases of GEJ and gastric adenocarcinomas in unusually young patients, marked by rapid progression despite standard first-line chemotherapy combined with trastuzumab and pembrolizumab. Although both tumors had high HER2 expression (IHC 3+), treatment failure occurred unexpectedly early, resulting in short overall survival. These cases highlighted aggressive tumor biology with intrinsic resistance to current HER2-directed therapies, which is likely a reflection of underlying gene fusions and amplifications. In both patients, comprehensive tissue genomic profiling revealed high-level *FGFR2* amplification at diagnosis with copy numbers of 128 and 241, respectively, in addition to other oncogenic alterations, including *MYC*, *CCNE1*, *ERBB2*, *BRAF*, and *TP53* mutations. These findings are consistent with the chromosomal instability (CIN) molecular subtype of gastric cancer, known for harboring multiple concurrent amplifications and exhibiting poor responses to therapy. *FGFR2* amplification, although found in 2-9% of gastroesophageal cancers, has been increasingly recognized as a biomarker of poor prognosis and therapeutic resistance, particularly when co-amplifications are present (5).

*FGFR2* fusions are exceedingly rare in gastric/GEJ cancers, reported in only 0.5–3% of cases (5). *WDR11*, a gene responsible for encoding a protein in the WD repeat-containing protein family (14), has been identified as a rare fusion partner with *FGFR2* in gastric cancer (15). Specific fusions involving *WDR11*-*AS1* are even less common, with limited cases described in the literature. For instance, a *WDR11*-*AS1*-*FGFR2* fusion was identified in a GEJ tumor at extremely low frequency in a prior study (16). The *WDR11-AS1* gene is a non-protein coding RNA gene that may have a role in gene expression and regulation of its sense counterpart, *WDR11*, although its exact mechanisms and cellular effects are not fully understood (14). Located in close proximity on chromosome 10q26, the *WDR11* and *FGFR2* genes may be susceptible to chromosomal instability in this region and predispose to genomic rearrangements (14, 17).

In our first case (46-year-old male with GEJ adenocarcinoma), molecular testing of both tissue and ctDNA identified *FGFR2* amplification (plasma copy number 83.1) and a *WDR11*-*AS1*–*FGFR2* fusion (2.4% VAF) was seen on ctDNA testing at disease progression. Despite subsequent enrollment in a clinical trial evaluating an FGFR inhibitor, the patient’s disease progressed rapidly, leading to death within nine months from diagnosis. In the second case (19-year-old female with gastric adenocarcinoma), a similar plasma profile was observed with *FGFR2* amplification (copy number 82.6) and a low-level *WDR11*-*AS1*–*FGFR2* fusion (0.1% VAF). This fusion was classified as lacking an active kinase domain; however, a reciprocal fusion (*FGFR2*–*WDR11*-*AS1*) could have been present but undetected. This patient also succumbed to disease within four months of diagnosis after a fulminant clinical decline.

The biological implications of *WDR11*-*AS1*–*FGFR2* fusions remain incompletely understood. However, fusions involving *FGFR2* generally result in constitutive receptor activation, bypassing ligand-dependent signaling and driving oncogenesis (1). This mechanism likely contributes to therapeutic resistance, including resistance to HER2-targeted therapies, as observed in both cases (18). Importantly, both patients had concurrent alterations in other signaling cascades, such as ERBB2, which likely explain tumor resistance to targeted treatments.

The complexity and heterogeneity of gastroesophageal cancers are increasingly recognized as major obstacles for optimizing therapeutic strategies. This heterogeneity encompasses spatial variability both within the primary tumor and metastatic sites of disease, as well as temporal changes that occur over time. Consequently, single-site tissue biopsies may incompletely capture the spectrum of molecular alterations which are crucial for decision-making. Liquid biopsy is emerging as a non-invasive approach to overcome these limitations and allows an opportunity to monitor tumor dynamics and evolving genetic changes in real-time (19). While it should not substitute tissue testing when feasible, the two cases presented above demonstrate that concurrent or sequential tissue and liquid NGS testing can provide additional benefit in guiding patient care. Given the rarity and complexity of *FGFR2* fusions, further clinical studies are needed to explore the efficacy of FGFR2 inhibitors and combination therapies, as well as to assess the role of liquid biopsy in detecting these alterations for therapeutic decision-making. There are already promising data with bemarituzumab, FGFR2b directed antibody, in the first line setting for advanced gastric and GEJ cancers with FGFR2b overexpression (10). As such, targeting this pathway in this disease is of significant interest. These cases also highlight the need to address clinical challenge of biomarker overlap. With a growing number of biomarker-based therapies, especially in the first line setting for advanced GEA, we will continue to see a growing number of patients whose tumors express more than one targetable alteration. Future clinical studies should focus on targeted therapeutics combination strategies, especially as the threshold positivity for clinical intervention is lowered with novel therapeutics, such as antibody drug conjugates and bispecific antibodies. These cases also highlight the critical need for comprehensive genomic profiling in advanced gastric and gastroesophageal cancers, especially in patients with early disease progression, as it may offer novel therapeutic opportunities and enhance personalized treatment approaches.

## Data Availability

The original contributions presented in the study are included in the article/supplementary material. Further inquiries can be directed to the corresponding author.
